# Complete assignment of the vibrational spectra of borazine: the inorganic benzene

**DOI:** 10.1039/c8ra04845b

**Published:** 2018-06-29

**Authors:** Stewart F. Parker

**Affiliations:** ISIS Facility, STFC Rutherford Appleton Laboratory Chilton, Didcot OX11 0QX UK stewart.parker@stfc.ac.uk

## Abstract

Borazine continues to be relevant in industries as diverse as energy utilisation *via* fuel cells and as a possible route to boron nitride. Despite it having been known for almost a century, the vibrational spectroscopy of borazine is still incomplete. The inclusion of inelastic neutron scattering spectra has enabled the observation of all of the internal modes of borazine (including the infrared and Raman forbidden modes) for the first time. A complete assignment has been generated with the use of dispersion corrected DFT calculations. This has shown that the accepted ordering of the modes is incorrect in some cases and rationalised conflicting assignments in the literature.

## Abstract 2

Inelastic neutron scattering spectroscopy has detected all of the internal modes of borazine, including the infrared and Raman forbidden modes.

## Graphical abstract

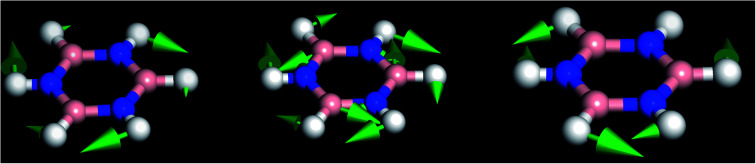

## Introduction

**Fig. 1:**
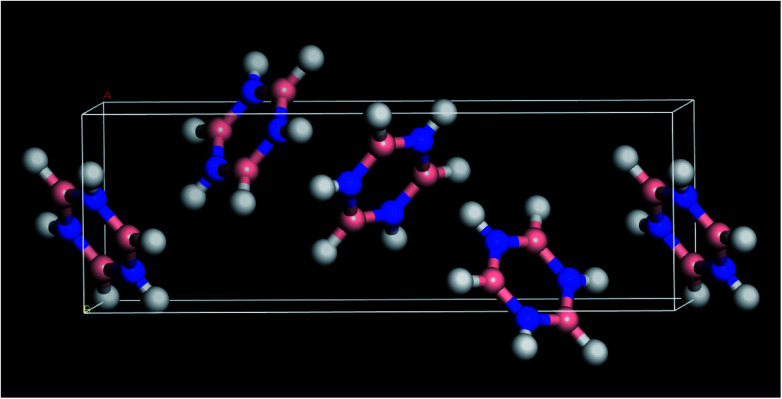
Solid state structure of borazine.^3^ Blue = nitrogen, pink = boron, white = hydrogen.

Borazine (1,3,5,2,4,6-triazatriborinane, B_3_N_3_H_6_, see Fig. 1 for the structure) was discovered in 1926 by Stock and Pohland^1^ as a product of the reaction of diborane and ammonia. It is isostructural and isoelectronic with benzene and, like benzene, is a colourless liquid under ambient conditions. The apparent similarity to benzene has led to borazine being called the ‘inorganic benzene’,^2^ although this view is not universally accepted.^3^

Borazine is of current interest for two reasons: boron nitride and hydrogen storage. It can be used as a carbon-free precursor to boron nitride, with the advantage that the initial condensation to polyborazylene results in a processable material.^4^ Borazine is also used in preparative routes using chemical vapour deposition.^5^ In the area of hydrogen storage materials, borazine is an unwelcome by-product^6^ of the dehydrogenation of ammonia borane because it rapidly poisons the low temperature fuel cells that are often the destination of the released hydrogen.

The vibrational spectroscopy of borazine has been investigated many times over the years. However, the work has almost exclusively been concerned with gas phase infrared studies.^7–11^ The molecule offers multiple possibilities for isotopic substitution: ^1,2^H, ^10,11^B and ^14,15^N and many of the possible combinations have been examined. Apart from a brief conference report of the solid state infrared spectrum,^12^ there are no reported solid state spectra. The molecule has been analysed by normal coordinate analysis using empirical force fields^13,14^ and also by an *ab initio* study.^15^ The analysis of the spectra is greatly complicated by the fact that the free molecule has *D*_3h_ symmetry (as shown by gas phase electron diffraction^16^) which results in three silent modes.

The purpose of the present work was to measure the vibrational infrared, Raman and inelastic neutron scattering (INS)^17^ spectra of the solid state for the first time and to assign these with periodic density functional theory calculations. The comparison of observed and calculated spectra has provided a complete assignment. INS spectroscopy has no selection rules, so all modes are allowed and the infrared and Raman inactive modes are observed for the first time.

## Experimental

Borazine was obtained from Gelest and used as received. All operations involving transfer of borazine were conducted in a glovebox to avoid hydrolysis. Infrared spectra (2 cm^−1^ resolution, 256 scans) were recorded between 105 K and 298 K with a Bruker Vertex 70 Fourier transform infrared spectrometer using a Specac single reflection variable temperature attenuated total internal reflection accessory. Raman spectra were recorded in the range 13–235 K with a previously described^18^ Renishaw InVia system using 785 nm excitation and have been corrected for the instrument response. In this configuration the largest Raman shift obtainable is ∼3200 cm^−1^. INS spectra were recorded at <10 K using the TOSCA^19^ and MAPS^20^ spectrometers at ISIS.^21^ For TOSCA, only the data from the backscattering detectors was used, because the neutron absorption from ^10^B in the natural abundance borazine resulted in no usable data in the forward scattering detectors.

Dispersion corrected periodic density functional theory (DFT-D) calculations were carried out using the plane wave pseudopotential method as implemented in the CASTEP code.^22,23^ Exchange and correlation were approximated using the PBE^24^ functional with the Tkatchenko–Scheffler (TS) dispersion correction scheme^25^ within the generalized gradient approximation (GGA). The plane-wave cut-off energy was 1000 eV. Brillouin zone sampling of electronic states was performed on 10 × 10 × 4 Monkhorst–Pack grid (30 *k*-points). The equilibrium structure, an essential prerequisite for lattice dynamics calculations was obtained by BFGS geometry optimization after which the residual forces were converged to ±0.00755 eV Å^−1^. Phonon frequencies were obtained by diagonalization of dynamical matrices computed using density-functional perturbation theory^26^ and also to compute the dielectric response and the Born effective charges, and from these the mode oscillator strength tensor and infrared absorptivity were calculated. In addition to the calculation of transition energies and intensities at zero wavevector, phonon dispersion was also calculated along high symmetry directions throughout the Brillouin zone. For this purpose, dynamical matrices were computed on a regular grid of wavevectors throughout the Brillouin zone and Fourier interpolation was used to extend the computed grid to the desired fine set of points along the high-symmetry paths.^27^ Transition energies (assuming the harmonic approximation) for isotopic species were calculated from the dynamical matrix that is stored in the CASTEP checkpoint file using the PHONONS utility.^28^ The atomic displacements in each mode that are part of the CASTEP output, enable visualization of the modes to aid assignments and are also all that is required to generate the INS spectrum using the program ACLIMAX.^29^ It is emphasised that for all the calculated spectra shown the transition energies have not been scaled.

## Results

**Fig. 2:**
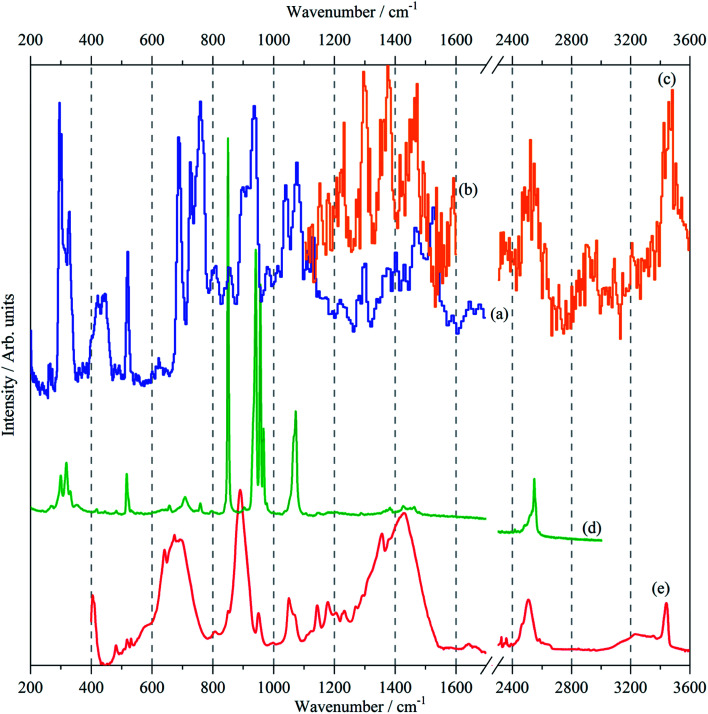
Vibrational spectra of borazine in the solid state. INS spectra recorded at 10 K with: (a) TOSCA (b) MAPS, (*E*_i_ = 3200 cm^−1^), (c) MAPS, (*E*_i_ = 4800 cm^−1^), (d) Raman recorded at 11 K and (e) infrared recorded at 105 K.

The vibrational spectra; infrared, Raman and INS, of borazine in the solid state are shown for the first time in Fig. 2. The complementarity of the three techniques is immediately apparent: modes that are strong in one technique are often weak or absent in the others. The transition energies are listed in Table 1. The mode numbering and descriptions are that of Niedenzu *et al.*^10^

**Table 1:** Observed and calculated modes of borazine in the solid state

Observed^a^ cm^−1^			CASTEP^b^ cm^−1^			Description
Infrared	Raman	INS	Average^c^	Range^d^	Sym^e^	
	303 w, 324 w, 338 w	296 s, 302 s, 310 s, br 327 s	326	49	20E′′	Out-of-plane ring deformation
406 m, 410 sh		399 m, br 421 m, br 443 m, br	412	21	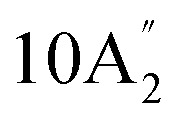	Out-of-plane ring deformation
517 vw, 522 vw, 530 vw	516 w, 523 sh	519 m	514	5	17E′	In-plane ring deformation
682 vs, vbr	710 w, br	689 s	698	25	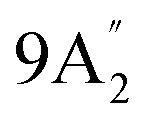	Out-of-plane N–H bend
	761 w	729 s, 758 s	747	54	19E′′	Out-of-plane N–H bend
	850 s	856 w	841	1	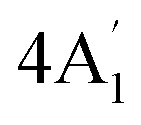	In-plane sym ring deformation
890 vs, br		910 br	883	14	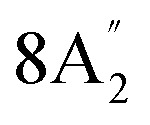	Out-of-plane B–H bend
			896	21	18E′′	Out-of-plane B–H bend
	932 sh, 942 s	935 s	918	9	15E′	In-plane B–H bend + N–H bend + B–N stretch
950 w	957 s		924	4	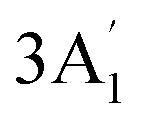	In-plane B–N sym stretch (ring breathe)
		1040 m	1020	14	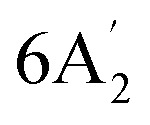	In-plane B–H bend
1050 m, 1069 w	1065 m, 1072 m	1078 m	1054	9	16E′	In-plane N–H + B–H bend + B–N stretch
		1220 w	1207	2	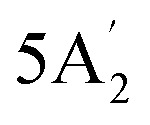	Asymmetric B–N stretch
		1297 m	1276	6	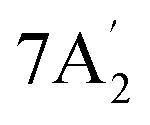	In-plane N–H bend
1356 w	1325 vw		1351	15	14E′	Asymmetric B–N stretch
1430 vs, vbr	1424 vw		1421	29	13E′	Asymmetric B–N stretch
2467 sh, 2506 m			2565	22	12E′	Asym B–H stretch
	2503 w, br 2547 w 2558 sh		2581	6	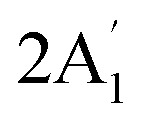	Sym B–H stretch
3421 w, sh			3445	2	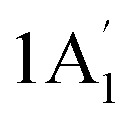	Sym N–H stretch
3442 m			3454	8	11E′	Asym N–H stretch

a s = strong, m = medium, w = weak, br = broad, sh = shoulder, v = very.

b Transition energies at the at the *Γ*-point of the complete unit cell containing four molecules.

c Average of the factor group split transition energies at the *Γ*-point.

d Difference between the highest and lowest transition energy of the factor group at the *Γ*-point.

e Mode number and symmetry label for the mode in *D*_3h_ symmetry.

**Fig. 3:**
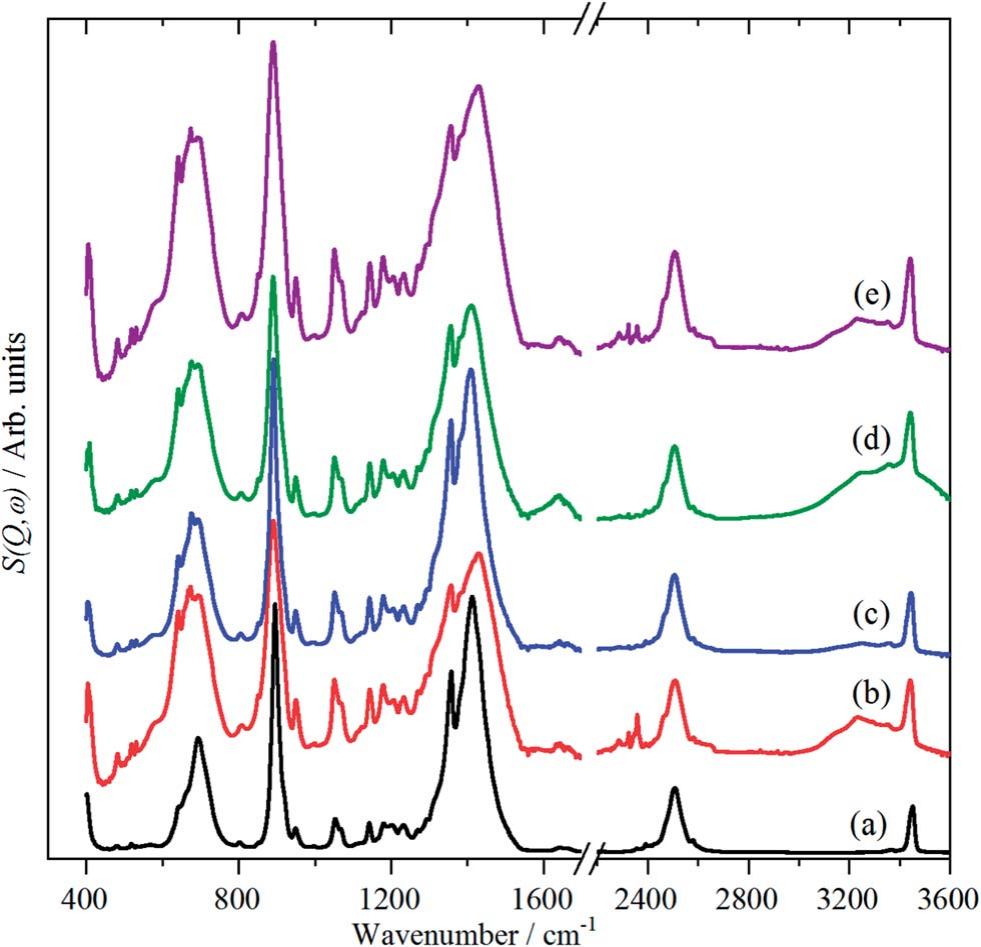
Vibrational temperature infrared spectra of borazine. (a) liquid at 298 K, solid at: (b) 258 K, (c) 213 K, (d) 160 K and (e) 105 K. The broad features at 1650 and 3300 cm^−1^ are due to ice.

**Fig. 4:**
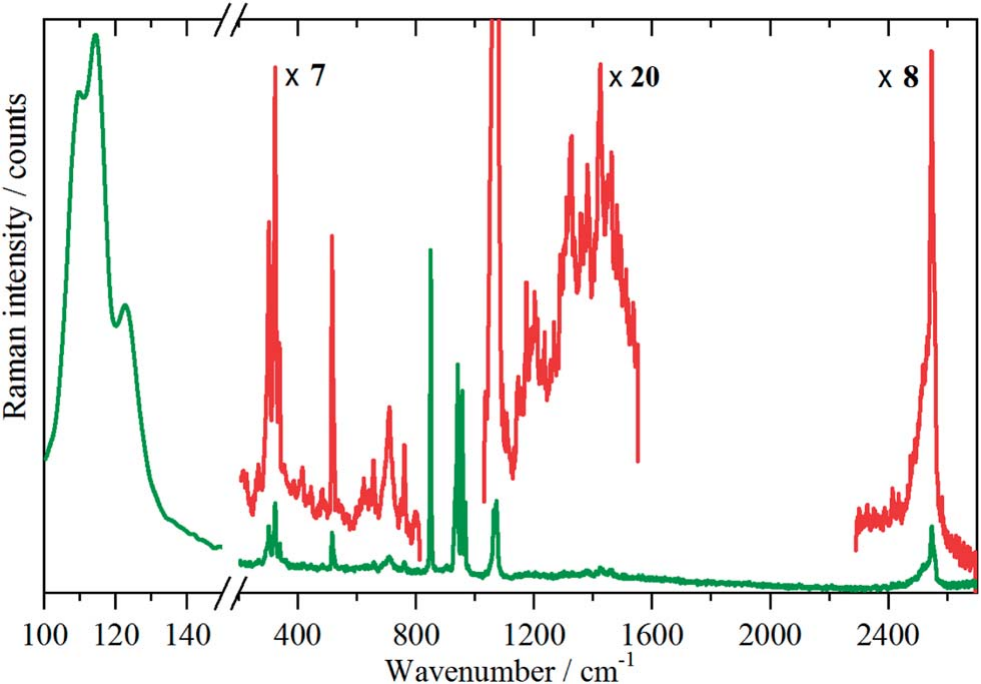
Raman spectrum of borazine in the solid state at 11 K.

Variable temperature infrared spectra, Fig. 3, show that the molecule is unchanged between 258 and 105 K, in agreement with the X-ray structure determination.^3^ Further the spectra are almost indistinguishable from that of the liquid state, Fig. 3a. The low temperature, 15 K, Raman spectrum allows a number of very weak bands to be observed, Fig. 4. The symmetric N–H stretch is most easily seen in the Raman spectrum. Unfortunately, with 785 nm excitation the CCD cuts-off at ∼3200 cm^−1^, consequently it was not observed. However, the infrared spectrum shows a shoulder at 3421 cm^−1^ on the main asymmetric stretch band. The DFT calculations (see later) show that the symmetric mode is activated in the solid state and the band at 3421 cm^−1^ is assigned to this mode.

**Fig. 5:**
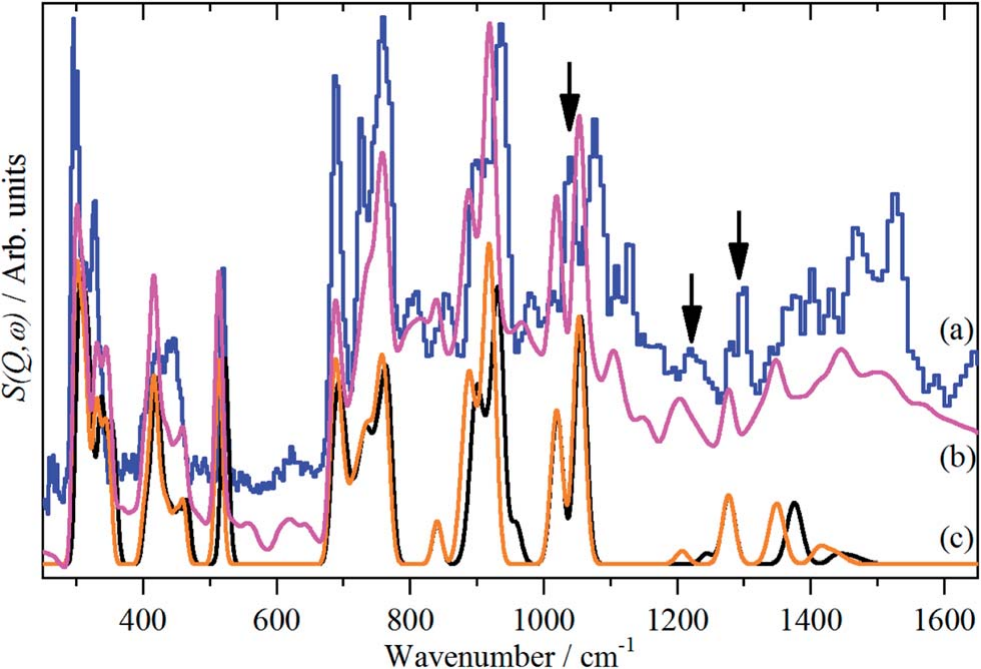
Comparison of the INS spectra of borazine in the solid state. (a) Experimental (TOSCA) and generated from the CASTEP calculation (b) complete unit cell, *Z* = 4, all ^11^B, all transitions and (c) fundamentals only for all ^10^B (black, rightmost trace) and ^11^B (orange, leftmost trace). The three infrared and Raman inactive 
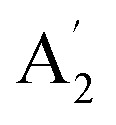
 modes are indicated by arrows.

The INS spectra, Fig. 2a–c and 5, show that the solid state is more complicated than the infrared or Raman spectra suggest. This is evidenced by the complex band shapes of the modes in the 300–500 and 700–800 cm^−1^ regions. INS spectra have no selection rules, hence all modes are allowed, however, there is a strong “propensity rule” that modes that involve proton motion will dominate the spectra.^17^ Further, INS spectra depend on both energy transfer, *ω* (cm^−1^), and momentum transfer, *Q* (Å^−1^), thus are able to access the entire Brillouin zone, not just the *Γ*-point *i.e*. *Q* ∼0, as for infrared or Raman spectroscopies. Thus the complex band shapes indicate that there is clearly significant factor group splitting and/or vibrational dispersion present in this system.

**Fig. 6:**
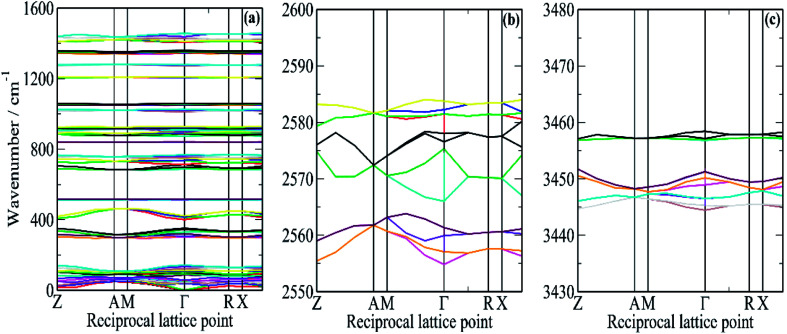
Dispersion curves of borazine in the solid state generated from the CASTEP calculation. (a) In the external mode and fingerprint region, (b) in the B–H stretch region and (c) in the N–H stretch region.

In order to better understand this system, periodic density functional theory (DFT) of the complete unit cell was employed. Borazine crystallises in the tetragonal space group, *P*4_3_2_1_2, with four molecules in the unit cell (*Z* = 4)^3^ and this was used as the initial structure for the computational study. A series of calculations, using a range of cut-off energies and *k*-point grids, always resulted in imaginary modes, indicating that the structure was dynamically unstable. The vibrational spectroscopy shows no evidence for a structural phase change, suggesting that the model was inadequate in some way. The problem was resolved by the inclusion of the Tkatchenko and Scheffler (TS)^25^ semi-empirical dispersion correction for DFT. This attempts to account for non-covalent forces, such as hydrogen bonding and van der Waals interactions in the solid state. With the inclusion of the TS correction, all real modes were obtained across the entire Brillouin zone, Fig. 6.

It can be seen that for the modes in the 300–500 and 700–800 cm^−1^ regions that there is both strong dispersion: the mode at ∼400 cm^−1^ at the *Γ*-point has a transition energy of ∼450 cm^−1^ at the *A* and *M* points in the Brillouin zone. There is also a large factor group splitting: columns 4 and 5 of Table 1 show the average and the range (defined as highest–lowest transition energy) of the factor group components.

Fig. 5a and b compares the observed and calculated INS spectra of borazine in the internal mode region. It can be seen that there is generally good agreement for both the transition energies and their intensities.

## Discussion

Unlike ammonia–borane, H_3_N–BH_3_,^30^ the structure shows no evidence for dihydrogen bonding, which is characterised by approximately linear N–H⋯H bonds and bent B–H⋯H bonds.^30^ In the geometry optimised structure the shortest H⋯H distance is 2.512 Å, outside the accepted range of 1.7–2.2 Å and larger than twice the van der Waals radius of hydrogen, 2.4 Å. Although borazine has a gas phase dipole moment of <0.1 D^16^ (and zero for the ideal *D*_3h_ structure), the calculations do show significant charge on the atoms: N: −0.92/−0.21, B: +0.52/+0.15, H(N): +0.42/+0.12 and H(B): −0.01/−0.06 electrons for the Mulliken and Hirschfeld charges respectively.

The good agreement between the observed and calculated spectra in Fig. 5, enables unambiguous assignments from the mode visualisations to be made. These are listed in Table 2 and compared to those in the literature. In *D*_3h_ symmetry, 
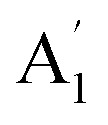
, E′ and E′′ are Raman active, 
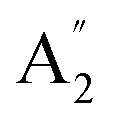
 and E′ are infrared active and 
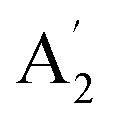
 are inactive in both, note that all modes are allowed in the INS spectrum. For the 
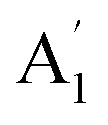
 and 
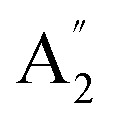
 modes there is general agreement on the assignments and this work confirms these. For the E′ modes, there is confusion over the ordering of modes 15 and 16. These are described as in-plane bending modes of B–H and N–H respectively.^10^ The mode animations show that these are in fact more complex and are coupled vibrations involving B–H and N–H bending as well as B–N stretching. On the basis of the relative amplitudes of the motion, mode 16 is assigned at 1078 cm^−1^ (N–H bend) and mode 15 at 935 cm^−1^ (B–H bend).

**Table 2:** Comparison of vibrational assignments of borazine in *D*_3h_ symmetry

Mode no.	Sym	This work		(Ref. 7) 1939	(Ref. 10 and 13) 1970	(Ref. 11) 1971	(Ref. 15) 1991	Description (method)^c^
		Calc.^a^	Obs.^b^					
1	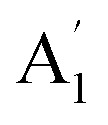	3475	3421	3450	3452	3488	3649	Sym N–H stretch (R)
2	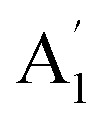	2553	2547	2535	2535	2545	2708	Sym B–H stretch (R)
3	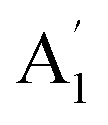	920	957	938	940	940	958	In-plane B–N sym stretch (ring breathe) (R)
4	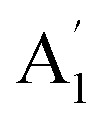	839	850	851	852	845	869	In-plane sym ring deformation (R)
5	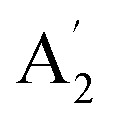	1207	1220	(1650)^d^			1332	Asym B–N stretch (INS)
6	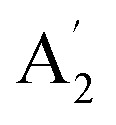	1020	1040	(1110)^d^		1195	1266	In-plane B–H bend (INS)
7	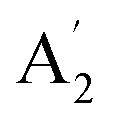	1276	1297	(800)^d^		782	1069	In-plane N–H bend (INS)
8	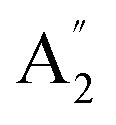	883	910	1098	917	913	943	Out-of-plane B–H bend (IR)
9	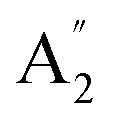	698	682	628	719	718	751	Out-of-plane N–H bend (IR)
10	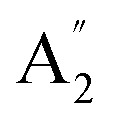	412	431^e^	415	394	403	396	Out-of-plane ring deformation (IR)
11	E′	3475	3442	3400	3486	3482	3652	Asym N–H stretch (IR)
12	E′	2543	2506	2519	2520	2513	2698	Asym B–H stretch (IR)
13	E′	1421	1430	1610	1465	1458	1524	Asym B–N stretch (IR)
14	E′	1351	1356	1466	1406	1394	1422	Asym B–N stretch (IR)
15	E′	918	935	917	1096	1102	1098	In-plane B–H bend + N–H bend + B–N stretch (INS)
16	E′	1051	1078	717	990	1068	963	In-plane N–H + B–H bend + B–N stretch (INS)
17	E′	514	519	525	518	518	523	In-plane ring deformation (INS)
18	E′′	896	890	1070	968	977	934	Out-of-plane B–H bend (IR)
19	E′′	747	758	798	798	770	727	Out-of-plane N–H bend (INS)
20	E′′	326	296	288	288	280	283	Out-of-plane ring deformation (INS)

a Average of the factor group split transition energies at the *Γ*-point.

b Transition energy of the strongest mode in the spectra closest in energy to the calculated value.

c Calculated from the force field, not observed.

d Method is the technique where the mode is best observed; INS (INS), Raman (R) or infrared (IR).

e This entry is the centre frequency of the complex line shape resulting from both dispersion and a large factor group splitting in this mode.

The assignment of the highest energy E′′ mode is also debated. It is usually assigned to the weak band at 967 cm^−1^. However, our calculations show no mode at this energy and the INS spectrum only shows a very weak mode. This is inconsistent with it being the B–H out-of-plane bend, which would have a large amplitude of motion of the proton and hence strong INS intensity. The present work locates this mode at 890 cm^−1^ (896 cm^−1^ calculated).

**Fig. 7:**
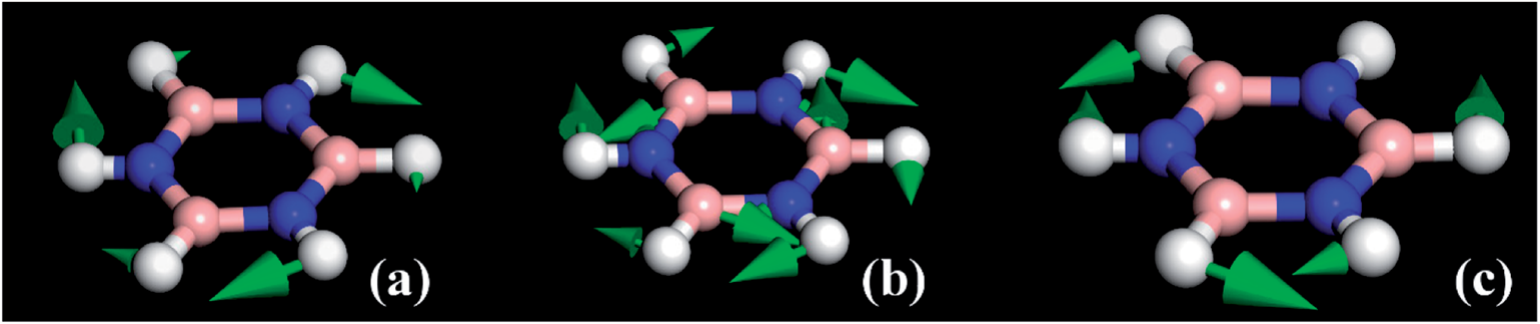
The atomic displacements for the modes calculated at (a) 1279, (b) 1215 and (c) 1026 cm^−1^.

The modes that were least certain are the in-plane 
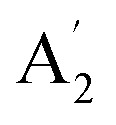
 modes, 5, 6 and 7, which are described as asymmetric B–N stretching, B–H and N–H bending respectively. Fig. 7 shows the atomic displacements for the calculated modes at 1279, 1215 and 1026 cm^−1^ respectively. It is apparent that these correspond to modes 7, 5 and 6.

The INS spectra, Fig. 2a–c and 5, provide the first experimental observation of the modes at 1220, 1040 and 1297 cm^−1^ for modes 5, 6 and 7 respectively. The only previous claim^11^ was by infrared spectroscopy of matrix isolated borazine. Two weak modes were seen which were believed to be combinations involving modes 6 and 7. This work shows that those assignments are incorrect.

Borazine containing natural abundance boron (20% ^10^B, 80% ^11^B) was used for this work. This leads to the question as to whether this is manifested in the spectra. In the gas phase and in a matrix, weak ^10^B satellite peaks are observed for some of the modes,^9,11^ I have shown previously how the isotopic distribution in a crystal may be calculated,^31^ and for natural abundance borazine with four molecules in the primitive cell, the largest constituents are: (^10^B^11^B^11^B, ^10^B^11^B^11^B, ^11^B^11^B^11^B, ^11^B^11^B^11^B) = 23.19% and (^10^B^11^B^11^B, ^11^B^11^B^11^B, ^11^B^11^B^11^B, ^11^B^11^B^11^B) = 20.62%, the all-^11^B species is only a minor constituent, 6.87%. Fig. 5c shows the calculated INS spectra for the most extreme compositions: all ^10^B and all ^11^B. It can be seen that the difference is small for most of the modes.

## Conclusions

Despite it having been known for almost a century, the vibrational spectroscopy of borazine was still incomplete. The inclusion of INS spectra has enabled the observation of all of the internal modes of borazine for the first time. A complete assignment has been generated with the use of DFT-D calculations. This has shown that the accepted ordering of the modes is incorrect in some cases and rationalised conflicting assignments in the literature.

## Conflicts of interest

There are no conflicts of interest to declare.

## Supplementary Material
